# Potential role of microRNA-7 in the anti-neuroinflammation effects of nicorandil in astrocytes induced by oxygen-glucose deprivation

**DOI:** 10.1186/s12974-016-0527-5

**Published:** 2016-03-09

**Authors:** Yin-Feng Dong, Zheng-Zhen Chen, Zhan Zhao, Dan-Dan Yang, Hui Yan, Juan Ji, Xiu-Lan Sun

**Affiliations:** Jiangsu Key Laboratory of Neurodegeneration, Department of Pharmacology, Nanjing Medical University, 140 Hanzhong Road, Nanjing, 210029 China; School of Nursing, Nanjing University of Chinese Medicine, Nanjing, China

**Keywords:** Nicorandil, miR-7, Neuroinflammation, Oxygen-glucose deprivation

## Abstract

**Background:**

It is generally recognized that the inflammatory reaction in glia is one of the important pathological factors in brain ischemic injury. Our previous study has revealed that opening ATP-sensitive potassium (K-ATP) channels could attenuate glial inflammation induced by ischemic stroke. However, the detailed mechanisms are not well known.

**Methods:**

Primary cultured astrocytes separated from C57BL/6 mice were subjected to oxygen-glucose deprivation (OGD); cellular injuries were determined via observing the changes of cellular morphology and cell viability. MicroRNA (miR) and messenger RNA (mRNA) level was validated by real-time PCR. The interaction between microRNA and the target was confirmed via dual luciferase reporter gene assay. Expressions of proteins and inflammatory cytokines were respectively assessed by western blotting and enzyme-linked immunosorbent assay.

**Results:**

OGD resulted in astrocytic damage, which was prevented by K-ATP channel opener nicorandil. Notably, we found that OGD significantly downregulated miR-7 and upregulated *Herpud2*. Our further study proved that miR-7 targeted *Herpud2* 3′UTR, which encoded endoplasmic reticulum (ER) stress protein-HERP2. Correspondingly, our results showed that OGD increased the levels of ER stress proteins along with significant elevations of pro-inflammatory cytokines, including tumor necrosis factor α (TNF-α) and interleukin 1β (IL-1β). Pretreatment with nicorandil could remarkably upregulate miR-7, depress the ER-related protein expressions including glucose-regulated protein 78 (GRP78), C/EBP-homologous protein (CHOP), and Caspase-12, and thereby attenuate inflammatory responses and astrocytic damages.

**Conclusions:**

These findings demonstrate that opening K-ATP channels protects astrocytes against OGD-mediated neuroinflammation. Potentially, miR-7-targeted ER stress acts as a key molecular brake on neuroinflammation.

## Background

Astrocytes are specialized and most numerous glial cell types in the brain, they play crucial roles in central nervous system (CNS) homeostasis [1]. Except for providing support to neurons under ischemia-hypoxic condition, astrocytes can also secrete a series of pro-inflammatory and anti-inflammatory cytokines to modify the ambient microenvironment [2, 3]. Post-ischemic inflammation mediated by astrocytes is a vital contributing factor in the CNS injury [4–10]. Brain ischemia induces damage to astrocytes and stimulates the release of pro-inflammatory cytokines, such as tumor necrosis factor-α (TNF-α) and interleukin-1β (IL-1β), which are crucial for the pathological processes of brain ischemic injury [11].

It has been revealed that ischemic injury could trigger endoplasmic reticulum (ER) stress, which subsequently inspires inflammatory response process and cellular damage [12, 13]. In mild ER stress, the unfolded-protein response (UPR) processes are activated, which is served to synthesis of new chaperons to refold the unfolded protein [13]. Consequently, this process could compensate damage. However, the intense and prolonged ER stress could activate apoptotic pathways, such as the upregulation of the CEBP homologous protein (CHOP) and the activation of pro-apoptotic protein-caspase-12, and thereby result in cell death. Our previous study revealed that ATP-sensitive potassium (K-ATP) channels were involved in cerebral ischemia injury and post-ischemic inflammation [14, 15], but the potential molecular mechanisms remain not well understood.

Recently, considerable evidences support microRNAs as an important regulatory molecule of astrocytic functions in ischemic stroke [16–19] and regarded them as potential candidates for stroke therapeutics. MicroRNAs are small molecule non-coding RNAs; various aspects of microRNAs as well as their targets have been reported. By the transcription and/or translation of protein-coding genes, microRNAs exert many biological functions such as regulating ER stress, inflammation, and apoptosis [20–22].

In the present study, we found that oxygen-glucose deprivation (OGD) elevated ER stress and inflammatory injuries in astrocytes. Our further study revealed for the first time that OGD decreased inflammation-associated microRNA 7 (miR-7) but increased the messenger RNA (mRNA) levels of Herpud2, one of the miR-7’s targets. Pretreatment with nicorandil could upregulate miR-7 and alleviate OGD-induced ER stress and inflammatory injuries in astrocytes. These results suggest that miR-7 may be a potential target for the anti-inflammation effect of nicorandil.

## Methods

### Cell cultures and treatment

Primary culture of astrocytes was prepared as previously described [23]. Cortices were aseptically separated from 2-day-old C57BL/6 mice pups, minced, and dissociated by trypsinization. Then, they were plated on T75 culture flasks in Dulbecco’s modified Eagle’s medium (DMEM) containing 10 % fetal bovine serum (FBS) and incubated at 37 °C in an incubator supplemented with 5 % CO_2_. After 14 days, the flasks were shaken for 1 h at 37 °C to remove the other glia. The astrocytes were then detached by trypsinization and plated in six-well plates at a density of 1 × 10^6^ cells/ml. In the drug-treated group, cells were pretreated with nicorandil (a K-ATP channel opener) (TOCRIS, Bristol, UK) for 1 h before OGD and persisted to reoxygenation 24 h. The control group was treated by the vehicle. The experimental groups were divided as follows: (A) control; (B) nicorandil (10 μM); (C) OGD; and (D) OGD plus nicorandil (10 μM). All experimental procedures were approved by IACUC (Institutional Animal Care and Use Committee of Nanjing University of Chinese Medicine).

### Oxygen-glucose deprivation and reoxygenation model

The protocol was previously described [15]. After washing twice, astrocytes were immersed in 1-ml deoxygenated custom DMEM without glucose and FBS (GIBCO, CA, USA). Then, they were placed inside an incubator (Thermo scientific, Waltham, MA, USA) for 5 h with a premixed gas (1 % O_2_, 94 % N_2_, 5 % CO_2_). After that, cells were immersed in normal DMEM containing 10 % FBS and transferred to a CO_2_ incubator (95 % air and 5 % CO_2_) for 24 h. For non-OGD group, cultures were incubated in DMEM containing 5.5-mM D-glucose (GIBCO, CA, USA) and 10 % FBS and placed in 5 % CO_2_ in air at 37 °C for 28 h.

### Cell viability analysis

The MTT assay was used to evaluate the cell viability as previously described [11]. Primary cultured astrocytes were seeded onto 96-well plates at a density of 5 × 10^4^ cells per well. After OGD 5 h and reoxygenation 24 h, the MTT (0.5 mg/ml) was dissolved in the cell medium and incubated at 37 °C for 4 h. Then, we used dimethyl sulfoxide (DMSO) to dissolve the MTT-formazan product. The absorbance was obtained by a Dynatech MR5000 plate counter at a test wavelength of 570 nm.

### Real-time PCR for mRNA

Total RNA was extracted with Trizol Reagent (Invitrogen, USA) following the manufacturer’s instructions; 20 million cells were used per milliliter of Trizol. Then, complementary DNA (cDNA) was synthesized from 1 μg total RNA in a 20-μl total volume containing 4-μl mRNA Reversed Transcription Kit (Takara 036A, Japan) and RNase-free water. Reaction was performed in a Thermal Cycler as follows: 42 °C for 15 min, 87 °C for 5 s, and 4 °C forever. PCR amplifications were performed on the ABI 7300 Sequence Detection System (Applied Biosystem, USA); they were performed in a total volume of 20 μl, containing 1 μl cDNA sample and 10 SYBR Green PCR Master Mix (Roche, UK). PCR amplifications were always performed in double wells, using the universal temperature cycles: 10 min at 95 °C, followed by 40 cycles consisting of 15 s at 95 °C and 1 min at 60 °C. The quantification was performed by the comparative Ct (cycle threshold) method, using the glyceraldehydes 3-phosphate dehydrogenase (GAPDH) as internal control. Primers (Sangon Biotech, Shanghai) used for real-time PCR are as listed in Table 1.

**Table 1:** The primers for PCR amplification

Gene name	Forward (5′ to 3′)	Reversed (5′ to 3′)
Herpud2 (mouse)	GGCCCAGTGCTGAATGAAGA	CAGCATGGCTCCCATTACCA
GAPDH (mouse)	CATGGCCTTCCGTGTTCCTA	CCTGCTTCACCACCTTCTTGAT
miR-7 RT (mouse)	AGCATTCGTCTCGACACAGCAACAAAATC	
MiR-7 (mouse)	TGACTCTGCTGGAAGACTAGTGAT	TAGAGCATTCGTCTCGACACAG
U6 RT (mouse)	AACGCTTCACGAATTTGCGT	
U6 (mouse)	CTCGCTTCGGCAGCACA	AACGCTTCACGAATTTGCGT
WT vector for Herpud2 3′UTR (mouse)	cacaactcgagTAAGCTTCTCATGCATATGA	aaggatccGGGCAAGATGCTACTAGCACA
Mut vector for Herpud2 3′UTR (mouse)	TACTGCAGAAGGAGCTTTATTCATTTCAATTATGTGTA	AGCTCCTTCTGCAGTACTGTTAGCAATGCTATGTTGTTAG

### Real-time PCR for miRNA

Total RNA was isolated with Trizol Reagent (Invitrogen, USA) following the manufacturer’s instruction. Reverse transcription was performed using the Takara MicroRNA ReverseTranscription Kit (Takara 037A, Japan). Total RNA (500 ng) was reverse-transcribed with 2 μl 5×PrimeScript Buffer, 0.5 μl PrimeScript RT Enzyme Mix, 1 μl Specific miRNA RT primer, RNA 1 μl, and 5.5 μl RNase-free water. Reverse transcription reaction was performed in a thermal cycler as follows: 42 °C for 15 min, 87 °C for 5 s, and 4 °C forever. PCR amplifications were performed on the ABI 7300 Sequence Detection System (Applied Biosystems, USA); they were performed in a total volume of 20 μl, containing 1 μl cDNA sample and 10 SYBR Green PCR Master Mix (Roche, UK). PCR amplifications were always performed in double wells, using the universal temperature cycles: 10 min at 95 °C, followed by 40 cycles consisting of 15 s at 95 °C and 1 min at 60 °C. The expression of miR-7 was normalized using U6 as the internal control. Measurements were normalized to U6 (ΔCt) and comparisons calculated as the inverse log of the ΔΔCT to give the relative fold change for miR-7 level. Primers (Shanghai GenePharma Co., Ltd, Shanghai) used for real-time PCR are listed in Table 1.

### Plasmids

The 3′UTRs of Herpud2 mRNA harboring the predicted miR-7a binding sequences were PCR amplified from mouse genomic DNA and cloned into Bam HI and Xho I of the pLUC-Report luciferase vector (Shenzhen Kangbio Biological Technology Co., Ltd, Shenzhen, Guangdong) to generate the Herpud2-3′UTR reporter construct. Mutagenesis of predicted targets with a mutation of 7 bp from the site of perfect complementarity was performed using a site-directed Mutagenesis Kit (Takara). The primers are listed in supplementary Table 1.

### Dual luciferase target validation assays

HEK 293T cells were plated at a density of 2 × 10^4^ cells/well in 96-well plates 1 day before transfection. When cells were grown to 50 % confluence in 96-well plates, they were co-transfected with 0.2 μg plasmid DNA, 0.15 μg sensor reporter gene, and 0.45 μg miR-7 mimics or miRNA negative control (NC) (Gemma Pharmaceutical Technology Co., Ltd, Shanghai) using Fugene (Roche) according to the manufacturer’s instructions. After 48 h, cells were lysed and assayed using dual luciferase reporter assay system (Promega) according to the manufacturer’s protocol. Results were displayed as relative luciferase activity, while Renilla luciferase activity was normalized to firefly luciferase activity.

### Western blotting

The cytosolic and nuclear proteins in samples were extracted according to the KEYGEN protein extraction kit (Nanjing, Jiangsu, China). Protein concentrations were determined using the Beyotime BCA Kit (Beyotime Biotechnology, Shanghai). The supernatants (30 μg protein) were separated by Tris-glycine SDS-PAGE, transferred to PVDF membranes (Millipore, USA) with the electrophoretic transfer system (Trans-blot Semi-dry Transfer Cell, Bio-Rad, Hercules, CA, USA), and blocked with 5 % nonfat dry milk in Tris-HCl buffer saline (TBS, pH 7.4) containing 0.1 % Tween 20 (TBS-T) for 1 h at room temperature. Then, the PVDF membranes were incubated with primary antibody against glucose-regulated protein 78 (GRP78) (1:1000, CST, Boston, USA), CHOP (1:1000, CST, Boston, USA), Caspase-12 (1:800, CST, Boston, USA), H3 (1:800, Bioworld Technology, USA), nuclear factor κB (NF-κB) (1:800, Bioworld Technology, USA), phosphorylated IκB kinase a/β (pIKK a/β) (1:800, CST, Boston, MA, USA), and glyceraldehyde-3-phosphate dehydrogenase (GAPDH) (1:1000, CST, Boston, USA) overnight at 4 °C. After being washed in TBS-T, the membranes were incubated with corresponding secondary antibody for 1 h at room temperature. Finally, visualization of the signal was performed by enhanced chemiluminescence (Ultra-Lum, Claremont, CA, USA). Quantification of bands was made by scanned densitometric analysis and Image J analysis system.

### Enzyme-linked immunosorbent assay

After 24 h reoxygenation, we collected the medium from astrocytes. Release of the pro-inflammatory cytokines (TNF-α and IL-1β) from the cellular supernatant was performed using specific enzyme-linked immunosorbent assays (ELISAs) (R&D Systems, UK) according to manufacturers’ guidelines.

### Statistical analysis

Data are shown as mean ± S.E.M. Unless stated otherwise, all statistical quantitative assessments were carried out and performed in a blinded manner: for two groups, paired *t* test, for three or more groups, one-way analysis of variance (ANOVA) followed by Student-Newman-Keuls tests. Differences were considered significant for *P* < 0.05.

## Results

### Nicorandil attenuates OGD-induced injury in astrocytes

**Fig. 1:**
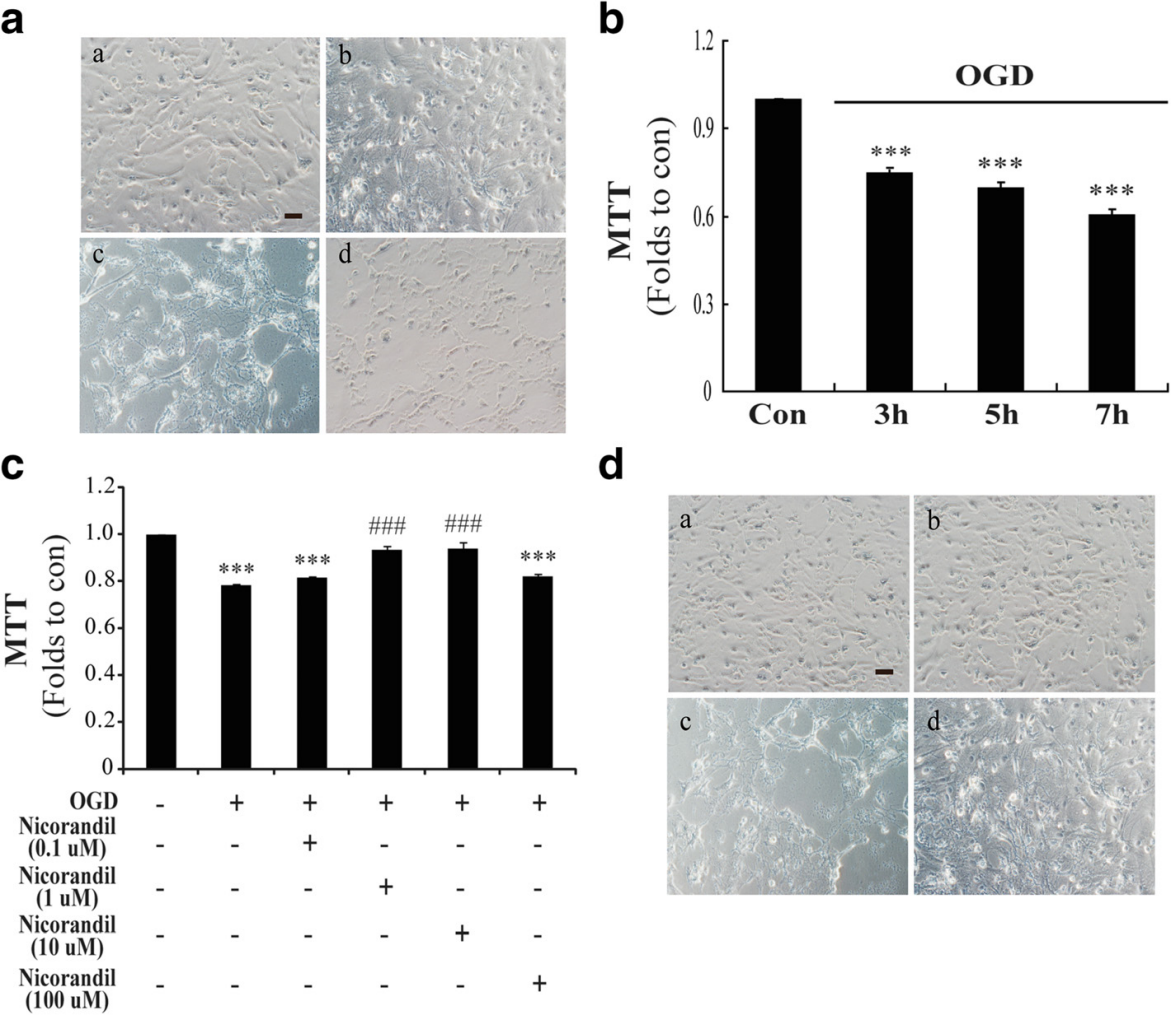
Nicorandil attenuates OGD-induced astrocytic injury. **A** The representative morphological alteration of astrocytes after different time-points of OGD. (*a*) Control, (*b*) OGD 3 h, (*c*) OGD 5 h, (*d*) OGD 7 h. *Scale bar*: 100 μm. **B** The time-course of cell viability changes induced by OGD in astrocytes. The changes of cell viability (**C**) and morphological alteration (**D**) of astrocytes pretreated with nicorandil. (*a*) Control, (*b*) nicorandil (10 μM), (*c*) OGD, (*d*) OGD plus nicorandil (10 μM). *Scale bar*: 100 μm. Data are represented as means ± S.E.M. from four independent experiments. ***P < 0.001 vs. control group; ^###^ *P* < 0.001 vs. OGD group

Normally, astrocytes are shaped by finely branched processes and have a small round body, as shown in Fig. 1A(a). Under OGD state, the appearance of astrocytes changed in a time-dependent manner. After OGD for 3 h and reoxygenation for 24 h, the appearance of some astrocytes displayed as large round body (Fig. 1A(b)) and the cell viability determined by MTT assay showed significant decrease (Fig. 1B). After OGD for 5 h and reoxygenation for 24 h, all of the astrocytes became reactive and displayed a stellated and hypertrophic with shorter processes and larger round cell body (Fig. 1A(c)), companied by a significant decrease of cell viability (Fig. 1B); When the OGD duration extended to 7 h, they exhibited disruption of cellular morphology and decrease in number (Fig. 1A(d)) with much more remarkable decrease of cellular MTT activity (Fig. 1B). However, pretreatment with nicorandil (1~10 μM) could attenuate OGD-induced decrease of cellular MTT activity and improved cellular morphology damaged by OGD-induced injury (Fig. 1D).

### MiR-7 targets the 3′UTR of *Herpud2*

**Fig. 2:**
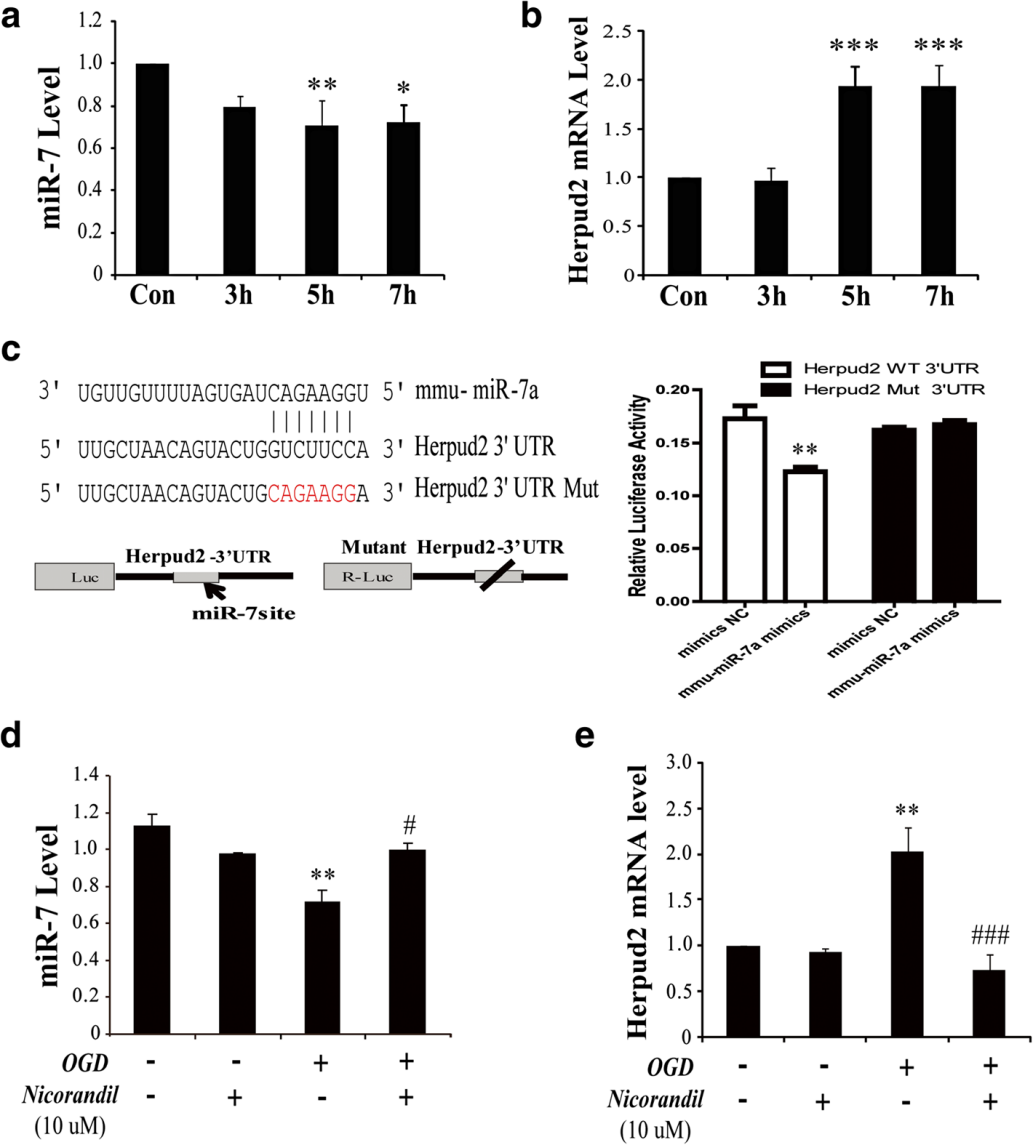
Nicorandil restrains OGD-induced upregulation of Herpud2 mRNA via miR-7. Time-course of OGD induced the changes of miR-7 (**a**) and Herpud2 mRNA (**b**) in astrocytes. **c** The target of miR-7 to Herpud2 3′UTR via Dual Luciferase Reporter Assays. Expression of miR-7 (**d**) and Herpud2 mRNA (**e**) pretreated by nicorandil. Data are represented as means ± S.E.M. from four independent experiments. *P < 0.05, **P < 0.01, ***P < 0.001 vs. control; ^#^ *P* < 0.05, ^###^ *P* < 0.001 vs. OGD group

MiR-7 is proved to be involved in cancer, diabetes mellitus, and Parkinson’s disease via targeting inflammation-related proteins. It was indicated by computational miRNA target prediction algorithms TargetScan (http://www.microrna.org) that miR-7 could potentially target the 3′UTR domain of *Herpud2* (HERP2), which was one of the HERP family members, acting as an important ER stress-related molecule. Thus, we determined the time-course of Herpud2 mRNA level and miR-7 via real-time PCR. Results demonstrated that expression of miR-7 in astrocytes was obviously downregulated by about 40 % since OGD for 5 h and reoxygenation for 24 h (Fig. 2a). Conversely, *Herpud2* mRNA showed significant increase (Fig. 2b). To validate whether miR-7 really targeted the 3′UTR of *Herpud2*, we constructed *Herpud2*-3′UTR reporter and used dual luciferase target validation assays to confirm the hypothesis. A construct containing the 3′UTR of *Herpud2* mRNA or the sequence with the mutant seed region was co-transfected along with miR-7 mimics or mimics NC into HEK293T cells. As shown in Fig. 2c, compared to mimics NC group, co-transfection of *Herpud2* WT 3′UTR with miR-7 mimics could significantly reduce the relative luciferase activity. Moreover, this targeting effect was specific because there was no significant change of the relative luciferase activity on condition of co-transfection of *Herpud2* Mut 3′UTR with miR-7 mimics (Fig. 2c). Therefore, these results demonstrated that miR-7 targeted the 3′UTR of *Herpud2*. To further study whether nicorandil affected expression of miR-7 and the target, we pretreated astrocytes with nicorandil and found that pretreatment with nicorandil (10 μM) could reverse OGD-induced decrease of miR-7 (Fig. 2d) and increase of *Herpud2* mRNA (Fig. 2e). These results implied that nicorandil protected against OGD-induced injury might be related to miR-7.

### MiR-7 alters expression of *Herpud2* and concerned with the protection of nicorandil against OGD-induced injury

**Fig. 3:**
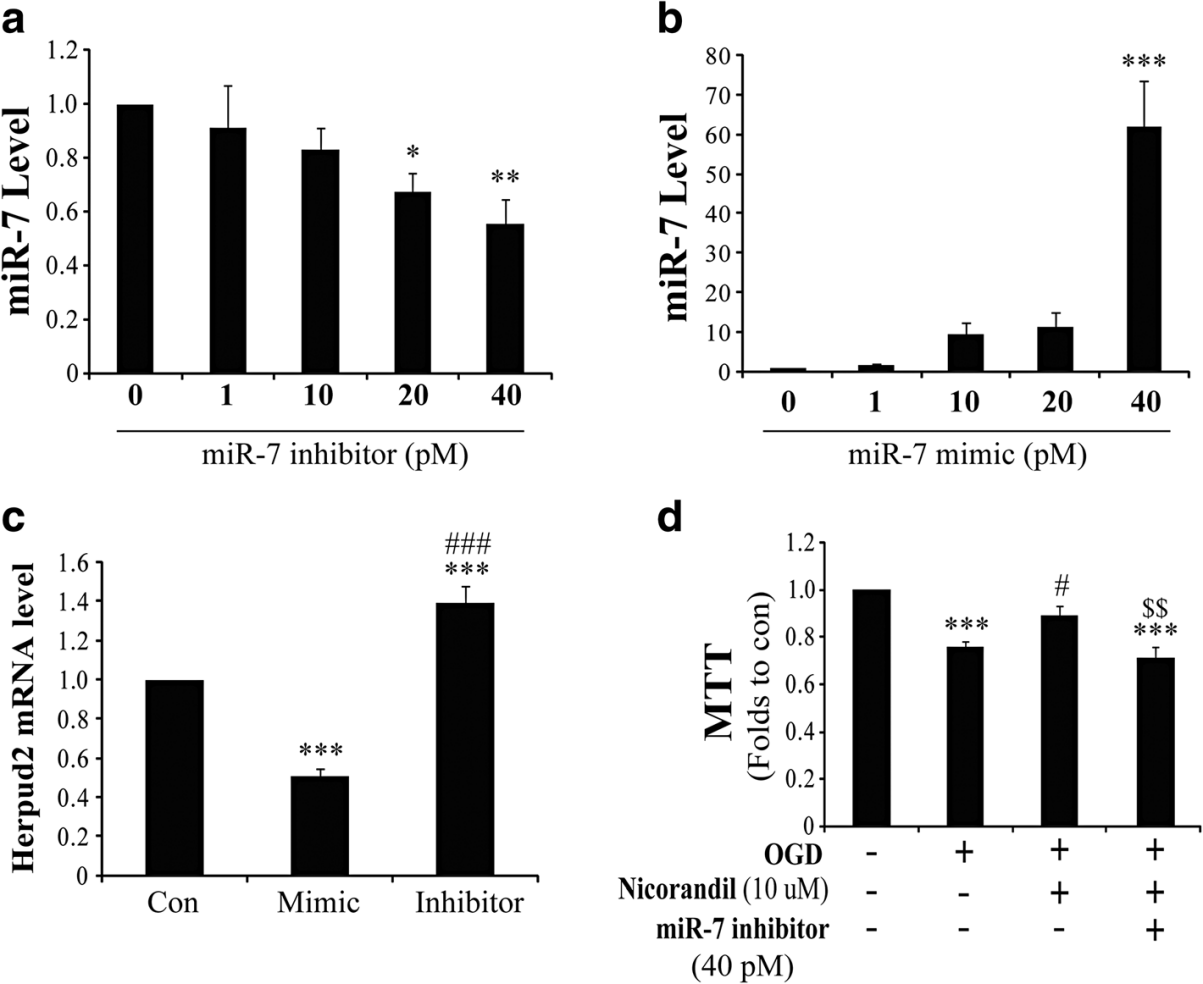
MiR-7 is indispensable for the protection of nicorandil against OGD-induced injury. Dose response of miR-7 level in primary cultured astrocytes after transfection with miR-7 mimic (**a**) and/or inhibitor (**b**). **c** Herpud2 mRNA level in primary cultured astrocytes transfected with miR-7 mimic and inhibitor. **d** Cell viability of astrocytes after pretreatment with nicorandil and (or) miR-7 inhibitor. Data are represented as means ± S.E.M. from four independent experiments. *P < 0.05, **P < 0.01, ***P < 0.001 vs. control; ^#^ *P* < 0.05, ^###^ *P* < 0.001 vs. OGD group; ^$$^ *P* < 0.01 vs. OGD plus nicorandil pretreatment group

To further verify the effects of miR-7, we established miR-7 mimic and inhibitor and transfected them into primary cultured astrocytes. We noted that transfection with miR-7 inhibitor or mimic could result in decrease or increase of miR-7. Transfection with 20- or 40-pM inhibitor could respectively induce downregulation of miR-7 by 33 and 45 % (Fig. 3a). While we used transfection with 40-pM miR-7 mimic, the result demonstrated that it could lead to an increase of about 62-fold in miR-7 level (Fig. 3b). Moreover, we also found that both transfection with miR-7 inhibitor (40 pM) and mimic (40 pM) altered expression of *Herpud2* mRNA (Fig. 3c); this was further evidenced when miR-7 regulated the expression of *Herpud2* mRNA. As we proved that pretreatment with nicorandil protected against OGD-induced injury and changed expression of miR-7, to further study whether the protection of nicorandil depended on miR-7, we transfected primary astrocytes with miR-7 inhibitor before pretreatment with nicorandil and detected cell viability by MTT assay. Interestingly, as shown in Fig. 3d, pretreatment with nicorandil (10 μM) could reverse OGD-induced decrease of cell viability; however, transfection with miR-7 inhibitor abolished this effect. Results indicated that nicorandil protected against OGD-induced injury depending on miR-7.

### Nicorandil depresses OGD-induced ER stress

**Fig. 4:**
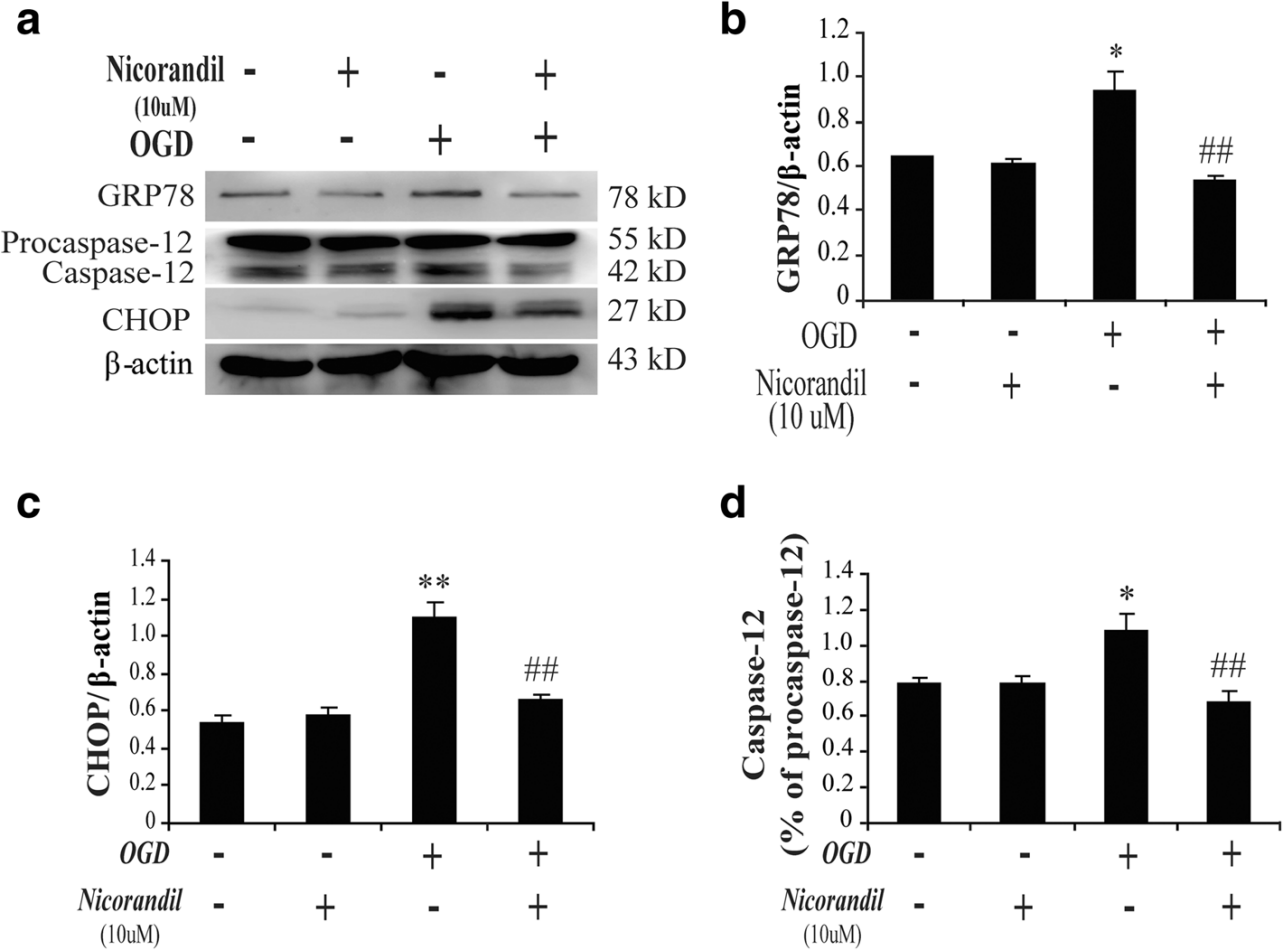
Nicorandil depresses OGD-induced endoplasmic reticulum stress. **a** Represented immunoblots of GRP78, CHOP, and Caspase-12. Immunoblotting analyses of GRP78 (**b**), CHOP (**c**), and Caspase-12 (**d**) in astrocytes. Data are represented as means ± S.E.M. from four independent experiments. *P < 0.05, **P < 0.01 vs. control group; ^##^ *P* < 0.01 vs. OGD group

Previous studies demonstrated that ER stress was crucial for brain ischemia injury. Our study proved miR-7 targeted the important ER stress relevant protein-HERP2, and nicorandil protected against OGD-induced injury depending on miR-7. To further verify whether nicorandil affected OGD-induced ER stress, we detected the expressions of three key important ER stress-related proteins, including molecular chaperon (GRP78/Bip), transcription factor (CHOP), and apoptosis-related protein (Caspase-12). Our results showed that GRP78, CHOP, and Caspase-12 were significantly upregulated in astrocytes after OGD (Fig. 4a–d). But pretreatment with nicorandil (10 μM) could inhibit OGD-induced upregulation of these ER stress proteins (Fig. 4a–d). These data confirmed that nicorandil could indeed depress OGD-induced ER stress.

### Nicorandil attenuates OGD-induced inflammatory responses

**Fig. 5:**
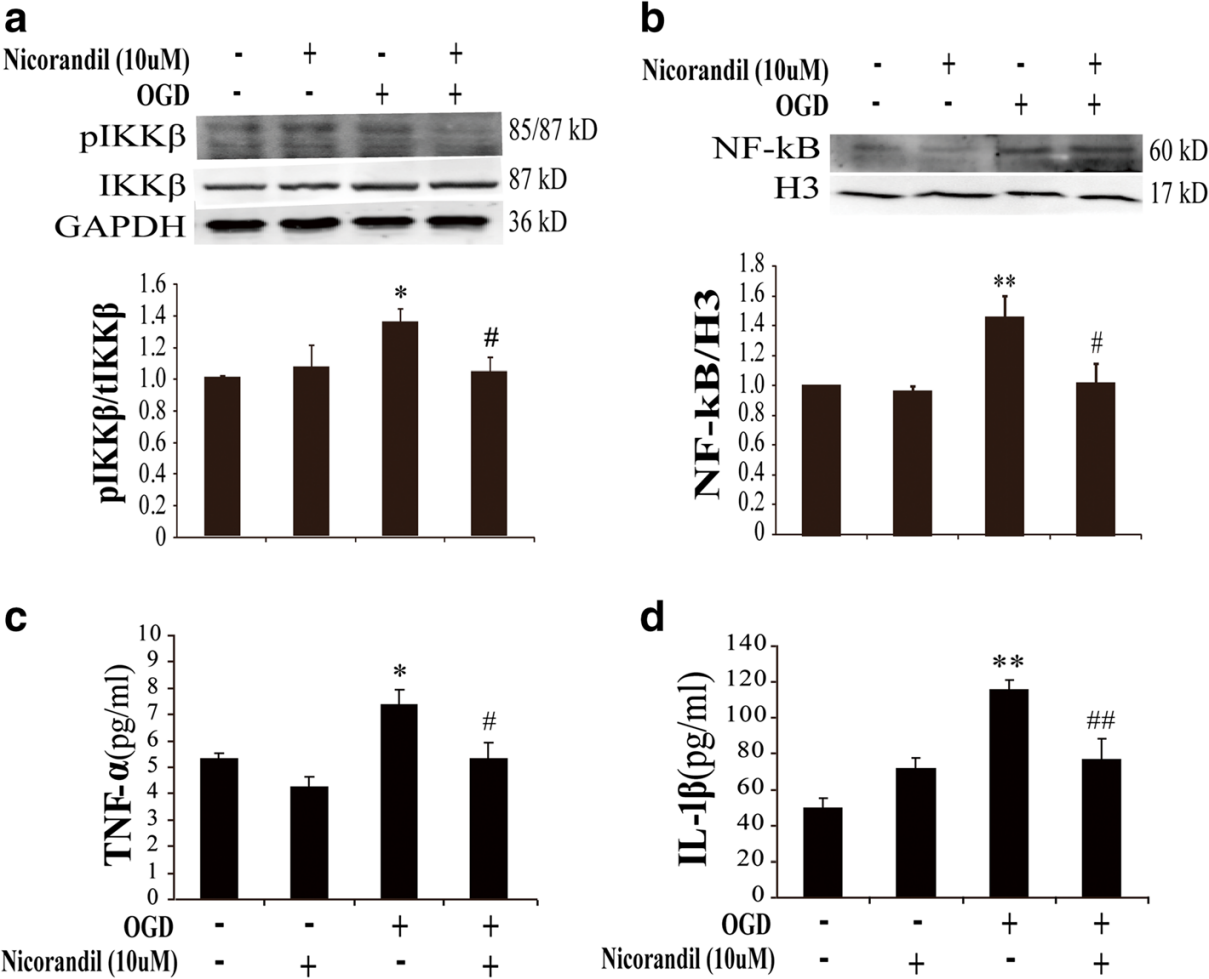
Nicorandil attenuates OGD-induced inflammatory responses via NF-κB signaling pathway. Represented immunoblots of phosphorylated IKKα/β (**a**) and nuclear NF-κB (**b**). Immunoblotting analyses of phosphorylated IKKα/β (**a**) and nuclear NF-κB (**b**). Data are represented as means ± S.E.M. from four independent experiments. *P < 0.05, **P < 0.01 vs. control group; ^#^ *P* < 0.05 vs. OGD group. ELISA assays of TNF-α (**c**) and IL-1β (**d**) in the supernatant, *n* = 8. *P < 0.05, **P < 0.01 vs. control group; ^#^ *P* < 0.05, ^##^ *P* < 0.01 vs. OGD group

ER stress easily stimulates inflammatory responses, both of them were crucial for brain ischemic injury. Therefore, we also observed the effect of nicorandil on OGD-induced inflammatory responses. As shown in Fig. 5, results displayed that OGD could upregulate the expression of phosphorylated IKKα/β (Fig. 5a) and nuclear NF-κB (Fig. 5b). Moreover, it could also increase the release of pro-inflammatory cytokines (TNF-α and IL-1β) to cell supernatant (Fig. 5c, d). However, pretreatment with nicorandil (10 μM) could reverse these effects; it could decrease OGD-induced upregulation of phosphorylated IKKα/β (Fig. 5a) and nuclear NF-κB (Fig. 5b) as well as reduced the content of pro-inflammatory cytokines including TNF-α (Fig. 5c) and IL-1β (Fig. 5d). Our research showed that nicorandil could attenuate OGD-induced inflammatory responses mediated by NF-κB signaling pathway.

## Discussion

Dysfunctions of reactive astrocytes are essential for the pathological processes of many CNS diseases, such as stroke, Parkinson’s disease, and Alzheimer’s disease [3, 24–26]. Accumulating studies suggest that ER stress and inflammation in astrocytes are important mechanisms involved in brain injury [27–29]. In the present study, our results also proved that both ER stress and inflammation are responsible for OGD-induced injury in astrocytes. Opening K-ATP channels could protect against OGD-induced inflammatory damage in astrocytes.

The endoplasmic reticulum is an important subcellular compartment and dispensable for cell survival; particularly, it plays an irreplaceable role in folding and processing cellular proteins. At the early stage of ER stress, unfolded-protein response (UPR) was initiated and demonstrated by accumulation of unfolded proteins including GRP78 and HERP [30]. It has been proved the pivotal roles of GRP78 in dysfunction of astrocytes during ischemic stroke [31], but little is mentioned about HERP. There are two types of HERP, such as HERP1 and HERP2, which are respectively encoded by Herpud1 and Herpud2 gene. HERP2 is constitutively expressed in cells, whereas HERP1 is highly induced by ER stress [32]. Both of them are essential for ER stress; they mainly participate in the process of ER-associated degradation and mediate ER stress-induced inflammation. Deficiency of HERP could attenuate ER stress-induced inflammatory reaction in atherosclerosis [33]. Most of the scientists focus on their functions but pay little attention to their regulation. MicroRNAs are proved to regulate gene expression at the post-transcriptional level, via degradation or translational inhibition of their target mRNAs [34]. In the present study, we found that miR-7 targeted the 3′UTR of Herpud2 (Fig. 2c). Our data showed that OGD could downregulate miR-7 (Fig. 2a) and upregulate mRNA level of Herpud2 (Fig. 2b); it motivated ER stress via upregulating ER stress proteins including GRP78, CHOP, and Caspase-12 (Fig. 4a–d). Thus, our study suggested that miR-7 might be an important upstream sign that modulate ER-associated degradation. MiR-7 is a proven small molecule that implicates in inflammation in various diseases [35], such as neurodegenerative disease and glioma [11, 36–38]. Our study revealed that miR-7 was also involved in OGD-induced ER stress and inflammatory responses in astrocytes.

ER stress is one of the most important acute-phase responses that mediate inflammatory responses. ER stress and inflammation interconnected through various mechanisms including activation of NF-κB signaling pathway [39]. The present data showed that OGD induced increase of pro-inflammatory cytokines including TNF-α and IL-1β. Inflammation induced by OGD was mediated by activating pro-inflammatory transcription factor NF-κB, which was the central mediator of pro-inflammatory pathway. Activated IKK phosphorylates inhibitor of κB, initiating inhibitor of κB and thereby leading to NF-κB activation, activated NF-κB then migrate to the nucleus, and facilitated transcription of pro-inflammatory cytokines such as TNF-α and IL-1β, which were transcribed by NF-κB [39]. In the present study, results demonstrated that OGD promoted production of TNF-α and IL-1β, which was mediated by phosphorylation of IKKα/β and activation of nuclear NF-κB. Opening K-ATP channel could inhibit OGD-induced inflammatory responses. These results suggested that K-ATP channels were an important regulator for OGD-induced inflammation.

The K-ATP channels widely express and act as an important sensor of energetic metabolism. Ischemia or OGD leads to cellular energy loss, which contributes to the dysfunction of K-ATP channels. In turn, dysfunction of K-ATP channels results in the intolerance of cells to stress. Particularly, K-ATP channels are abundant in the endoplasmic reticulum; dysfunction of them is responsible for ER stress pathway [40]. Beyond that, it is proved that K-ATP channels are pivotal for CNS inflammation, especially in glia. K-ATP channel knockdown increased glial reaction and production of inflammatory cytokines in ischemic brain [14]. K-ATP channel-deficient mice showed more susceptible to infection [41]. All of these suggest that K-ATP channels are pivotal for ER stress and inflammation. In the present study, pretreatment with nicorandil (a K-ATP channels’ opener) could rescue the reduction of miR-7 induced by OGD (Fig. 2d). Meanwhile, nicorandil could attenuate ER stress (Fig. 4) as well as decrease pro-inflammatory cytokine (TNF-α and IL-1β) production (Fig. 5). These results suggested that K-ATP channel’s opener alleviated OGD-mediated ER stress and inflammation via regulating miR-7 and thereby protected astrocytes against OGD-induced damage.

## Conclusions

Our data revealed that nicorandil protected against OGD-induced neuroinflammation damages in astrocytes. Further studies showed for the first time that miR-7 targeted the 3′UTR of *Herpud2*, which might be crucial for OGD-induced ER stress and inflammation in astrocytes.

## Abbreviations

CHOP: C/EBP-homologous protein
CNS: central nervous system
DMEM: Dulbecco’s modified Eagle’s medium
ER: endoplasmic reticulum
ELISA: enzyme-linked immunosorbent assay
EGFR: epidermal growth factor receptor
FBS: fetal bovine serum
GAPDH: glyceraldehyde-3-phosphate dehydrogenase
GRP78: glucose-regulated protein 78
IL-1β: interleukin-1β
K-ATP: ATP-sensitive potassium
LDH: lactic dehydrogenase
miR: microRNA
NF-κB: nuclear factor κB
OGD: oxygen-glucose deprivation
pIKKa/β: phosphorylated IκB kinase a/β
TNF-α: tumor necrosis factor-α
UPR: unfolded-protein response
